# A Hybrid Deep CNN Model for Abnormal Arrhythmia Detection Based on Cardiac ECG Signal

**DOI:** 10.3390/s21030951

**Published:** 2021-02-01

**Authors:** Amin Ullah, Sadaqat ur Rehman, Shanshan Tu, Raja Majid Mehmood, Fawad, Muhammad Ehatisham-ul-haq

**Affiliations:** 1 Software Engineering Department, University of Engineering and Technology Taxila, Punjab 47050, Pakistan; amin.ullah@uettaxila.edu.pk (A.U.); ehatishamuet@gmail.com (M.E.-u.-h.); 2 Center for Research in Computer Vision Lab (CRCV Lab), College of Engineering and Computer Science, University of central Florida (UCF), Orlando, FL 32816, USA; 3 Engineering Research Center of Intelligent Perception and Autonomous Control, Faculty of Information Technology, Beijing University of Technology, Beijing 100124, China; sadaqat.rehman@namal.edu.pk; 4 Department of Computer Science, Namal Institute, Mianwali 42250, Pakistan; 5 Information and Communication Technology Department, School of Electrical and Computer Engineering, Xiamen University Malaysia, Sepang 43900, Malaysia; rajamajid@xmu.edu.my; 6 Telecommunication Engineering Department, University of Engineering and Technology Taxila, Punjab 47050, Pakistan; engr.fawad@students.uettaxila.edu.pk

**Keywords:** electrocardiogram signal, arrhythmia, classification, 2D CNN, MIT-BIH, arrhythmia database

## Abstract

Electrocardiogram (ECG) signals play a vital role in diagnosing and monitoring patients suffering from various cardiovascular diseases (CVDs). This research aims to develop a robust algorithm that can accurately classify the electrocardiogram signal even in the presence of environmental noise. A one-dimensional convolutional neural network (CNN) with two convolutional layers, two down-sampling layers, and a fully connected layer is proposed in this work. The same 1D data was transformed into two-dimensional (2D) images to improve the model’s classification accuracy. Then, we applied the 2D CNN model consisting of input and output layers, three 2D-convolutional layers, three down-sampling layers, and a fully connected layer. The classification accuracy of 97.38% and 99.02% is achieved with the proposed 1D and 2D model when tested on the publicly available Massachusetts Institute of Technology-Beth Israel Hospital (MIT-BIH) arrhythmia database. Both proposed 1D and 2D CNN models outperformed the corresponding state-of-the-art classification algorithms for the same data, which validates the proposed models’ effectiveness.

## 1. Introduction

Cardiovascular issues are currently the primary cause of human morbidity, causing more than 17 million deaths each year. The World Heart Federation report witness about three fourth of the total cardiovascular disease (CVD) patients reside inside low-income regions across the globe [1]. Electrocardiogram (ECG) records the electrical activity generated by heart muscle depolarizations, which propagate in pulsating electrical waves towards the skin. Although the electricity amount is, in fact, very small, it can be picked up reliably with ECG electrodes attached to the skin (in microvolts, or uV) [2]. ECG signals contain no less than two critical pieces of statistics, including correlated to biomedicine’s healthiness [3,4,5] and associated with personal credentials or biometrics [6,7,8]. As a result of its easiness, several ECG categorizations processes have been established, counting manuals methods [9,10] and machine learning approaches [11,12,13,14,15,16]. The manual process is complicated. It is used for transient signals like ECG, often necessary for machine learning procedures with excessive computer assets. For better classification accuracy, machine learning methods are preferred compared to manual processes, though, a useful algorithm needed to diminish it.

One of the most common cardiovascular conditions is arrhythmias, where the heartbeats pattern deviates from its routine. These irregular patterns require classification into their subclasses; this information can be used to precisely suggest cure the patients. The ECG is widely used to diagnose and predict the irregular pattern of the human heart to diagnose cardiological diseases. The analysis of arrhythmia is primarily liable on the ECG. It is a significant current medical instrument that can record cardiac excitability, the process of transmission, and recovery. ECG is an essential and reliable diagnostic tool in modern medicine—the reliable automation of the interpretation of ECG signals is extremely beneficial for clinical routine and patient safety. Arrhythmia is an issue that deals with the irregular activity and pattern of the human heart [17].

Artificial intelligence, especially the machine learning technique, is one of the most useful tools in the prediction and diagnosis [18,19,20,21,22] of different types of fatal diseases, specifically cardiovascular diseases. Its results even sometimes perform better than medical experts [23]. Different types of medical data in the form of medical records, usually used for medical purposes in various hospitals. Moreover, the medical data can be regenerate quickly for different types of machine learning algorithms for various purposes. The first machine is trained on the given data; then, it learns from the provided data. After training, the device can detect and classify a patient, whether it is healthy or not, by visualizing the different attributes of patient records. Hence, in this way, a machine discovers the patterns in the given records that generally a human being cannot discover, because of deficiency of time or a deficiency of view.

Many approaches have been used to perform the arrangement of ECG signals like k-nearest neighbors (KNN), support vector machines (SVM), neural networks (NN), decision trees, linear discriminant analysis (LDA), Bayesian classifiers, etc. SVM is one of the best algorithms of machine learning technique (supervised classifier) [24,25], which is used in the taxonomy of the ECG wave in the discovery of Arrhythmia disease. The combination of SVM and LDA to classify six types of arrhythmia is presented in Reference [26]. An efficient classification model, which is based on the NN and Machine Language Program (MLP), gives better performance as compared to other feature extraction methods [27]. Deep Learning, such as Artificial Neural Networks (ANN), is successfully applied in applications, such as information retrieval [28], image recognition, object tracking, and language processing [29]. In Reference [30], a two-dimensional convolution neural network (2D CNN) model is developed to classify the ECG signals for the diagnosis of arrhythmia. However, in Reference [30], only a 2D CNN method is applied, while the 1D CNN model is completely ignored. The whole objective of Deep Learning is to solve problems characterized by high dimensionality, which have no rules.

**Figure 1:**
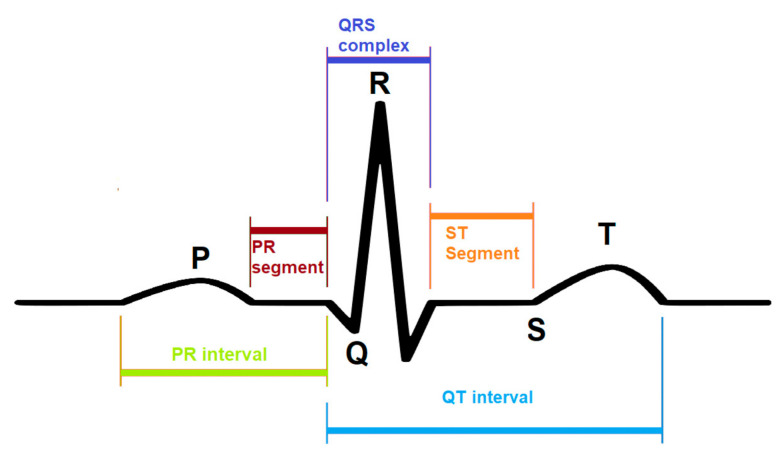
A model of an ECG signal. A different portion of the ECG signal is labeled to identify the functionality.

CNN is used in many studies to isolate the best characteristics from the ECG wave and analyze the extracted features for various determinations, i.e., detection of QRS wave, ST segment, as shown in Figure 1 or classification of a heartbeat. 1D CNN is trained to extract the best features from the ECG signal then classified these characteristics into five various types of arrhythmia [31]. To remove noise from the wave, it uses the wavelet method. The output layer is the last layer of the CNN model. It is also called a fully connected layer, contains five neurons that classify five different types of arrhythmias.

In the same way, the 1D CNN model is used to classify four various types of arrhythmia [32]. It also removes noise from the ECG signal in the pre-processing step, and then the denoised signal is input to the CNN model. The softmax layer is the last layer of the model, which gives four different outputs.

In the last few years, different pattern recognition techniques have been used in the prediction and classification of arrhythmia disease [33,34,35,36,37,38,39]. Typically, these methods have three main necessary steps; (i) pre-processing; (ii) feature extraction (iii) classification. Initially, the ECG wave is cleaned by eliminating the different types of noise and outliers, such as muscle contraction, baseline wanders, and power line interference using different algorithms [40,41,42,43,44,45,46,47].

In this research paper, a 1D and 2D CNN model are applied on 1D and 2D ECG signal, which classified five (N (normal), VPC (Ventricular premature complexes), LBB (left bundle branch), APC (atrial premature contraction), and RBB (right bundle branch)) and eight (NOR (normal), VFW (ventricular flutter wave), PVC (premature ventricular contraction), VEB (ventricular escape beat), RBB (right bundle branch block beat), LBB (left bundle branch block beat), PAB (paced beat) and APC (atrial premature contraction)) different types of arrhythmias, respectively. At last, the contributions of the proposed model are as follows.

- The suggested technique did not demand the post-processing of the ECG signal.
- It does not need handcrafted feature extraction.
- The proposed model has lower computational complexity than the previous models used to classify arrhythmia types.

It has better performance accuracy than the state-of-the-art algorithms for the arrhythmia classification.

## 2. Materials and Methods

### 2.1. Proposed System for Arrhythmia Classification Using 1D CNN

**Figure 2:**
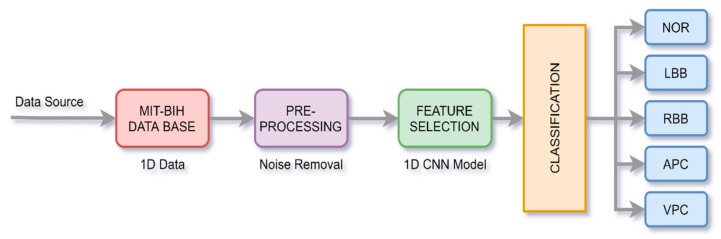
Process of ECG signal classification.

There are three crucial steps in the whole process, i.e., signal pre-processing, extraction of features from the data, and its classification. The overall process is shown in Figure 2.

#### 2.1.1. Pre-processing of Data

Pre-processing is a significant and essential part of applying machine learning techniques that are used to completeness and remove noise from the dataset. These processes are used to perform an eloquent examination of the data, and last it provides an optimum result. Firstly, we can apply the method of wavelet threshold and reconstruction algorithm of wavelet decomposition together to eliminate the noise from the original ECG wave. The technique of wavelet threshold can reduce electromyography noise, as well as power line noise interference. In contrast, the reconstruction algorithm of wavelet decomposition reduces the baseline drift noise from the noisy ECG signal. These two essential methods are initially used to eliminate noise from the ECG wave so that the wave can be used for further processing. Finally, the heartbeat signal as input data is put forward directly for the CNN model so that the best features are extracted, and ECG signals can be classified.

#### 2.1.2. 1D CNN Architecture

**Figure 3:**
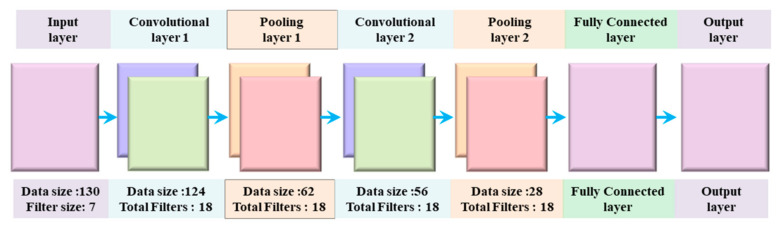
Proposed 1D CNN Model Architecture.

There are two main parts of CNN, extraction of features, and classification of features. The feature extraction part of CNN is responsible for the automatic extraction of the best characteristics from the ECG wave. These extracted characteristics are used for the accurate categorization of the ECG signal. In other words, these two parts of CNN accomplish the primary function of CNN [47]. The features extraction part of the CNN consists of a convolution layer and sampling layer. The fuzzy filter of the convolution layer is used to diminish the noise from the ECG wave, and then features of the ECG signal can be enhanced. The convolution process is done between the upper layer feature vector and the current layer convolution kernel layer. The activation function of CNN finally completes convolution process calculations. The overall architecture of the proposed 1D CNN is visualized in Figure 3 and outline in Table 1.

#### 2.1.3. Cost Function

The efficiency of training a neural network is apparent using a cost function, which signifies the proportion between the training sample and the gained output. The cost function is controlled by exploiting the optimizer function. There are various kinds of cost functions, though we use the cross-entropy function for loss measurement. To bound the cost function, along with the learning rate, a gradient descent-based optimizer function is applied.

(1) $$Z=\frac{-1}{m}\sum \left[x\ln a+\left(1-x\right)\ln\left(1-\beta \right)\right]$$

where *m* is the number of training data, *x* is an expected value, and *β* is an actual value from the output layer.

### 2.2. Method for Arrhythmia Classification Using 2D CNN

**Figure 4:**
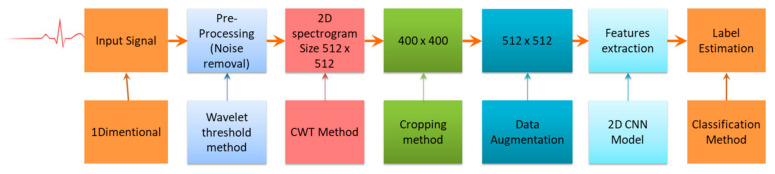
Complete procedures in ECG signal classification.

The accuracy obtained for arrhythmia classification through the 1D CNN model will be improved by transforming this 1D ECG signal into 2D ECG images, which is given as input to the 2D CNN model. The architecture of the proposed 2D CNN model is given in Figure 4. The whole process contains four steps, i.e., signal processing, generation of 2D images, augmentation of data, extraction of features from the data, and its categorization. The overall procedures are shown in Figure 4.

#### 2.2.1. Pre-Processing

The pre-processing step converts 1D ECG signal into 2D images, which are given to the 2D CNN model as input to isolate different characteristics contained in the data. Feature extraction helps in classifying various ECG types. This research study transforms a single 1D ECG beat wave into a single 512 × 512 grey-scale image. ECG waves of the MIT-BIH database are sliced into unique ECG beats based on Q-wave peak time and then also labeled with arrhythmia type on the same criterion. Due to this reason, the first and last 20 ECG waves before and after the Q-signal peak waves are chopped, and 2D images of ECG are defined by considering the Q-wave peak time. We use Equation (2) to determine the range of each ECG wave.

##### Generation of 2D Images

The one-dimensional convolutional neural network is mostly used for 1D signal, which suffers from the issue of flexibility. To resolve such issues, we have transformed the input signals to a two-dimensional form. The 2D CNN models are less prone to errors and can adopt the 2D kernels to represent time series data. Moreover, a vast set of robust methods available for the distinctive representation of 2D data. The 2D CNN can be used to capture the microstructural detail of the input data. The ECGs are converted in the 2D spectrogram by adapting the Continuous wave Transform method. The non-stationary details with varying instantaneous frequency can efficiently be represented in the frequency domain of continuous wavelet transform (CWT). The CWT can describe the frequency along with the localized amplitude of the time-varying signals. To describe such signals, we assume the signal to be stationary for the period of temporal window of finite support. The transformation of 1D signal into 2D (512 × 512) images is presented in the following expressions.

(2) $$X_{CWT}\left[r,\;c\right]=\overset{M-1}{\underset{j=0}{\sum }}x\left[j\right]h\left[r-j\right]e^{-i2\pi nj/M}$$

where *M* represents the window length, $x\left[j\right]$ denote the input signal. The log values of $X_{CWT}\left[r,\;c\right]$ represents as spectrogram (512 × 512) images.

#### 2.2.2. Augmentation of Data

When we supplement additional relevant information to our base data, this process is called augmentation. It reduces the human effort required in adding other data and improves the quality of data sometimes. This step might be helpful when 2D images are used as input to CNN models. The effectiveness of data augmentation can vary for different models. Some previous works achieved better results with augmentation, while some underwent a decrease in performance.

#### 2.2.3. Architecture of 2D CNN Model

This article uses the CNN model to classify ECG signals, which was introduced in 1989 to recognize handwritten zip codes. Due to the increase in free parameters of the raw image, the existing feed-forward networks are not feasible to classify 2D images. But embedding of multiple nonlinear filters in CNN models has made it possible to extract various local features of 2D images by correlating spatially adjacent pixels.

**Figure 5:**
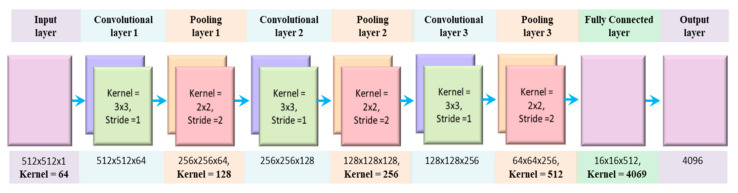
The proposed architecture of 2D CNN model.

Since it is feasible to extract and filter the spatial vicinity of 2D images through convolution and down-sampling layers, therefore this research introduces the transformation of 1D ECG signal into 2D ECG images. This scheme increases the classification accuracy of ECG signals to a level of not less than obtained via physician inspection. Hence, 2D CNN model can replace the traditional diagnosis process of ECG arrhythmia through eye vision inspection by medical experts. The architecture of the proposed model is given in Figure 5, and an explanation of different layers is shown in Table 2.

The following steps are incorporated to make the algorithm work more accurately.

#### 2.2.4. Activation Function

The activation function plays a crucial part in the classification process by assigning kernel size and weighting the output of the CNN model [48]. Nonlinear activation functions like leakage rectified linear units (LReLU), rectified linear units (ReLU), and exponential linear units (ELU) have gained popularity in recent days. ReLU activation function is amongst the commonly used activation functions [49]. It is approximately used in all CNN algorithms to set all negative values equal to zero. These zero settings inhibit several nodes from participating in the learning process. Other functions, like LReLU and ELU, which provide small negative values, are rarely used in classification techniques. ReLU activation function exhibits better results than LReLU activation function in arrhythmia classification; that is why we have employed it in our classification model. The given equation can mathematically denote the ReLU activation function.

(3) $$ReLU\left(x\right)=max\left(0;x\right)$$

#### 2.2.5. Cost Function

The cost function represents the difference between the targets and estimated labels. The optimizer function is employed to reduce the gap. Various cost functions are used in the neural network; however, the cross-entropy function the most frequently is used.

(4) $$C=\frac{-1}{n}\overset{N}{\underset{c=1N}{\sum }}\left(\left[y_{c}*l_{n}\left(a_{c}\right)+\left(1-y_{c}\right)l_{n}\left(1-a_{c}\right)\right]\right)$$

where *C* represent the cost function that is desired to be minimized. The $y_{c}$ is the target value, c is the class index. N is the total number of classes and a is true value. A gradient descent with a learning rate of 0.001 is used as an optimization method. CNN model and it reached the optimal point in less iteration.

Where *m* is the number of training data, x is an expected value, and *β* is an actual value from the output layer. Gradient descent is used as an optimizer function with a learning rate to reduce the error of cost function. Adagrad [50], Adam [51], and Adadelta [51] are famous optimizer functions. However, we used the Adam optimizer function in our CNN model, because in our experiments, it reaches optimal points quickly.

#### 2.2.6. Validation of Data

Validation of data will determine any model which gains maximum accuracy with the training data. If we did not use the validation data technique, then the model will fall into the problem of overfitting. Broadly, the loss value of the CNN model is the validation standard. Moreover, based on our notice, in different arrhythmia ECG signal classification, maximum sensitivity will not be received if we have stopped the CNN model according to lose value. Hence, we place the sensitivity mean of the validation data as the validation standard. We halt the learning technique when the weighted sensitivity means is no more increasing, and the evaluation of test data starts.

## 3. Experiments and Results

### 3.1. Data Set

The MIT-BIH database consists of 48 thirty minutes portions or pieces of 2-channel ambulatory ECG records, received from 47 patients analyzed by the BIH Arrhythmia Laboratory from 1975 to 1979. Twenty-three recordings were selected at random from a range of 4000 24-h ambulatory ECG recordings composed of a diverse inhabitant of indoor patients (60 out of 100) and outdoor patients (40 out of 100). The persist twenty-five recordings were chosen from a similar set to contains significant, nevertheless, clinically vital arrhythmias that will not be good symbolize in a less simple example. These recordings were digitized at 360 samples/sec per channel with an 11-bit determination over a series of 10 mV. Independently more than two cardiologists explain each record; dispute solves to get the computer-readable situation explanations for each record (about 110,000 interpretations) are contained in this database [48].

### 3.2. Experimental Procedure

The proposed CNN classifier is implemented in one of the most essential and famous machines-learning languages, Python, with an open-source library Tensor Flow developed by Google for deep learning. To train the CNN model, it needs a lot of computational power and training time. Therefore, we strongly recommended the Graphical processing unit (GPU) system to decrease the learning time for the CNN model.

### 3.3. Performance Evaluation

The proposed model diagnoses and classifies the following five different types of arrhythmia, i.e., N (normal), VPC (ventricular premature contraction), LBB (left bundle branch block), APC (atrial premature contraction), and RBB (right bundle branch block) with final accuracy 97.38%.

The precision-recall is a vital measurement technique used to assess recovery accuracy.

(5) $$A=\frac{T_{P}+T_{N}}{T_{P}+T_{N}+F_{P}+F_{N}}$$

where *T_p_* denotes the number of cases rightly classified as needed, *F_p_* represents the number of cases wrongly classified as needed, TN denotes the number of cases rightly classified as not needed and *F_N_* represents the number of cases wrongly classified as not needed.

## 4. Discussions of Both Models

**Figure 6:**
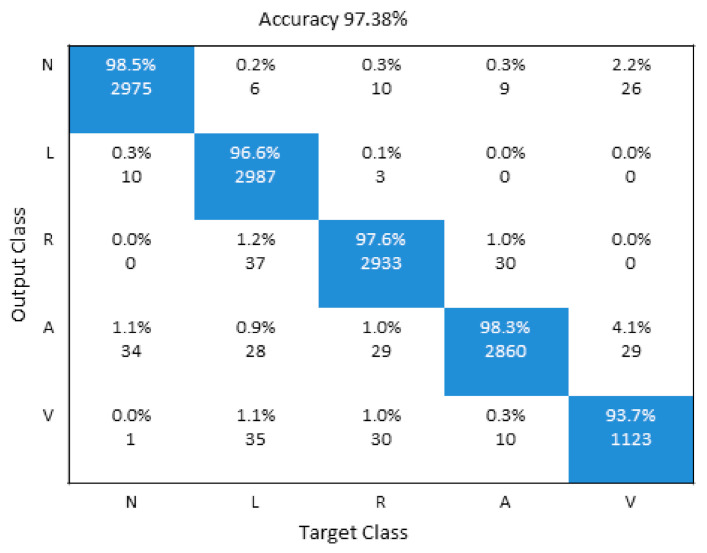
Illustration of confusion Matrix of 1D CNN model.

**Figure 7:**
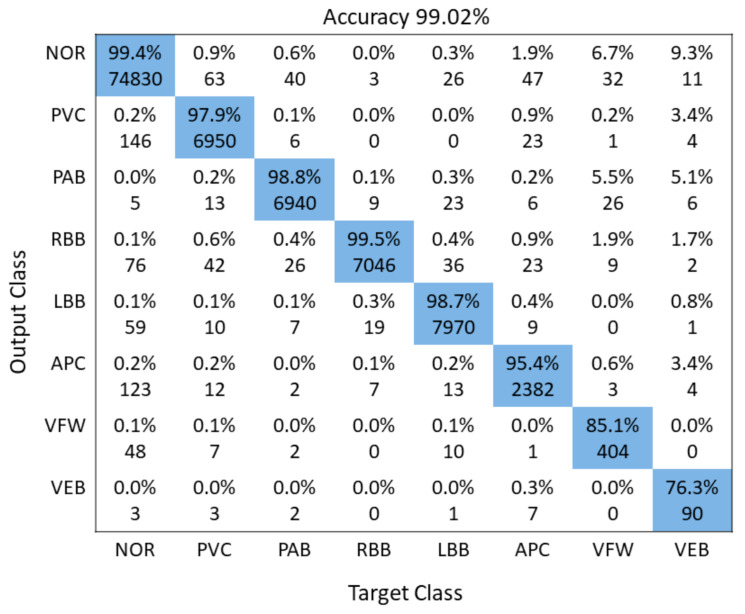
Illustration of confusion Matrix of 2D CNN model.

A detailed performance comparison between the proposed 1D CNN and 2D CNN models is presented using confusion matrices for five and all eight classes, respectively. The diagonal elements show the correctly classified classes, whereas anything off-diagonal represents an incorrect classification. For the 1D ECG and 2D ECG data used in experiments, results are presented for the proposed 1D CNN model (Figure 6) and the proposed 2D CNN model (Figure 7). The average accuracy of these three models is presented by averaging the diagonal values.

### 4.1. 1-D CNN Comparison with Other Algorithms

Table 3 shows the contrast between the optimized 1D CNN. It should be noted that there are various methods that used 1D data directly to classify arrhythmia [52,53,54,55,56,57,58]. Among these methods, SVM models achieved a lower classification accuracy (Reference [11]—89.72% and Reference [54]—86.40%) when compared with the proposed model. We also used 1D ECG signals as input to the 1D CNN model used in experiments and achieved a classification accuracy of 97.38%. The features extraction part of the 1D CNN consists of a convolution layer and sampling layer. The fuzzy filter of the convolution layer is used to diminish the noise from the ECG wave, and then features of the ECG signal can be enhanced. The convolution process is done between the upper layer feature vector and the current layer convolution kernel layer. The activation function of CNN finally completes convolution process calculations. Therefore, the proposed 1D CNN proved effective in classifying arrhythmia ECG signals, compared with other state-of-the-art techniques.

### 4.2. 2-D CNN Comparison with Other Algorithms

Table 4 displays the performance evaluation results of the proposed CNN algorithm with other CNN algorithms. Our proposed 2D CNN model gained 99.02% average accuracy. Recently, 2D CNN models have been employed to categorize the input ECG signals into their respective classes. The input 1D ECGs are transformed into 2D before the feature extraction process [16]. The 2D CNN models offer more distinctiveness and robustness towards noise in the input signals. The proposed model employs the CWT to convert the input 1D ECG into a 2D signal before extracting robust features. The proposed 2D CNN model offers better accuracy than the FFNN [61], moreover, our proposed model can categorize more classes as compare to FFNN. The proposed model consisting of three convolutional layers, followed by three pooling layers and a fully-connected layer, is lower in-depth than the AlexNet model. The VGGNet model consisting of ten convolution layer requires more computational resources than our proposed 2D CNN model. The DenseNet and ResNet are deeper networks that require more computational resources in comparison to our model.

In Table 4, we have compared the accuracy of our proposed model with that of recently reported works. The table shows our model outperforms most state-of-the-art model in term of classification performance. The classification accuracy of our model is 99.02% better than the techniques mentioned in References [11,57,62,63,64,65,66,67,68,69].

## 5. Conclusions and Future Work

Accurate categorization of ECG waves is very supportive in the prevention and diagnosis of CVDs. Deep CNN has proved to be very operative in amplifying the accuracy of diagnosis algorithms in the fusion of medicine and modern machine learning technology. Noise removal in source ECG signals using the Wavelet algorithm enhances the quality of the original ECG signal, improves the feature selection process, and hence, reduces the classification error. Our suggested 1D CNN model applied to the ECG signal, which learns useful attributes from the given data. Finally, it classifies the ECG signal automatically based on the extracted features. Using a 1D CNN model for categorizing the ECG signal provides an accuracy of 97.38%, which is better than the other algorithms used in previous works. Likewise, implementing efficient and practical techniques to classify arrhythmia ECG signal is achievable by transforming 1D ECG signal into 2D ECG images, and then using this as an input to the 2D CNN algorithm. Our proposed technique can 99.02% accurately classify arrhythmia by employing 2D images. This performance is a clear indication of the effectiveness of the proposed 2D CNN model to classify arrhythmia with transformed 2D ECG images.

In future work, we will design an integrated system to classify arrhythmia ECG signals, which will monitor and scan the patient’s ECG via the internal camera of the robot and will predict and diagnose the arrhythmia ECG signal to advise the medical expert. The current research relies on the use of a single ECG signal. The use of multi-channel data for categorizing ECG data will be useful in the future.

## Figures and Tables

**Table 1:** The architecture of the proposed 1D CNN Model.

Layer #	Type	Kernel Size	Stride	# Kernel
1	1D Conv	5	1	128
2	Pooling	2	2	-
3	1D Conv	5	1	128
4	Pooling	2	2	-
5	Fully Connected	-	-	520
6	Output Layer	-	-	5

**Table 2:** The architecture of the proposed 2D CNN model.

Layer #	Type	Kernel Size	Stride	# Kernel	Input Size
1	2D Conv	3 × 3	1	64	512 × 512 × 1
1	Pooling	2 × 2	2		512 × 512 × 64
2	2D Conv	3 × 3	1	128	256 × 256 × 64
2	Pooling	2 × 2	2		256 × 256 × 128
3	2D Conv	3 × 3	1	256	128 × 128 × 128
3	Pooling	2 × 2	2		128 × 128 × 256
4	2D Conv	3 × 3	1	512	64 × 64 × 256
4	Pooling	2 × 2	2		64 × 64 × 512
5	Fully Connected			4096	16 × 16 × 512
6	Output Layer			8	4096

**Table 3:** Comparison with other state-of-the-art algorithms. The proposed method is compared with different approaches on the same benchmark dataset (MIT-BIH arrhythmia database).

S. No.	Reference	Year	#Class	Methods	Accuracy
1	[52]	2007	5	NN	96.70%
2	[11]	2008	6	SVM	89.72%
3	[53]	2015	5	1D-CNN	95.14%
4	[54]	2016	5	1D-CNN	92.60%
5	[55]	2017	5	PNN	92.80%
6	[56]	2018	5	1D-CNN	90.00%
7	[57]	2019	5	1D-CNN	93.60%
8	[58]	2011	5	SVM, GA	97.30%
9	[59]	2013	5	NN, SVM	93.00%
10	[60]	2014	5	SVM	86.40%
11	Proposed Technique	2020	5	1D-CNN	97.38%

**Table 4:** Summarized performance evaluation results of different deep learning models on the MIT-BIH arrhythmia database.

No.	Reference	Year	#Class	Methods	Accuracy
1	[11]	2008	6	SVM	91.67%
2	[61]	2009	4	FFNN	96.94%
3	[62]	2009	5	PCA, ANN	98.30%
4	[63]	2010	3	LS-SVM	95.82%
5	[64]	2012	3	RFT	92.16%
6	[65]	2016	5	1D-CNN	96.40%
7	[66]	2018	2	DWT, DNN	98.33%
8	[67]	2018	5	2D-CNN	96.05%
9	[68]	2018	2	KNN	98.40%
10	[69]	2019	5	2D CNN	97.42%
11	[57]	2019	7	1D CNN	93.60%
12	Proposed Technique	2020	8	2D-CNN	99.02%
